# High Electrochemical Performance Silicon Thin-Film Free-Standing Electrodes Based on Buckypaper for Flexible Lithium-Ion Batteries

**DOI:** 10.3390/ma14082053

**Published:** 2021-04-19

**Authors:** Oyunbayar Nyamaa, Duck-Hyeon Seo, Jun-Seok Lee, Hyo-Min Jeong, Sun-Chul Huh, Jeong-Hyeon Yang, Erdenechimeg Dolgor, Jung-Pil Noh

**Affiliations:** 1 Department of Energy and Mechanical Engineering and Institute of Marine Industry, Gyeongsang National University, 2 Tongyeonghaean-ro, Tongyeong 53064, Korea; n.oyuka1231@yahoo.com (O.N.); dudug17@gnu.ac.kr (D.-H.S.); no9192@gnu.ac.kr (J.-S.L.); hmjeong@gnu.ac.kr (H.-M.J.); schuh@gnu.ac.kr (S.-C.H.); 2 Department of Mechanical System Engineering, Gyeongsang National University, 2 Tongyeonghaean-ro, Tongyeong 53064, Korea; jh.yagi@gnu.ac.kr; 3 School of Engineering and Applied Science, National University of Mongolia (NUM), P.O. Box 46A, Ulaanbaatar 14201, Mongolia; Erdenechimeg@seas.num.edu.mn

**Keywords:** flexible Li-ion battery, high capacity, oxidized MWCNTs, heated amorphous silicon, freestanding electrode, DC magnetron sputtering

## Abstract

Recently, applications for lithium-ion batteries (LIBs) have expanded to include electric vehicles and electric energy storage systems, extending beyond power sources for portable electronic devices. The power sources of these flexible electronic devices require the creation of thin, light, and flexible power supply devices such as flexile electrolytes/insulators, electrode materials, current collectors, and batteries that play an important role in packaging. Demand will require the progress of modern electrode materials with high capacity, rate capability, cycle stability, electrical conductivity, and mechanical flexibility for the time to come. The integration of high electrical conductivity and flexible buckypaper (oxidized Multi-walled carbon nanotubes (MWCNTs) film) and high theoretical capacity silicon materials are effective for obtaining superior high-energy-density and flexible electrode materials. Therefore, this study focuses on improving the high-capacity, capability-cycling stability of the thin-film Si buckypaper free-standing electrodes for lightweight and flexible energy-supply devices. First, buckypaper (oxidized MWCNTs) was prepared by assembling a free stand-alone electrode, and electrical conductivity tests confirmed that the buckypaper has sufficient electrical conductivity (10^−4^(S m^−1^) in LIBs) to operate simultaneously with a current collector. Subsequently, silicon was deposited on the buckypaper via magnetron sputtering. Next, the thin-film Si buckypaper freestanding electrodes were heat-treated at 600 °C in a vacuum, which improved their electrochemical performance significantly. Electrochemical results demonstrated that the electrode capacity can be increased by 27/26 and 95/93 μAh in unheated and heated buckypaper current collectors, respectively. The measured discharge/charge capacities of the USi_HBP electrode were 108/106 μAh after 100 cycles, corresponding to a Coulombic efficiency of 98.1%, whereas the HSi_HBP electrode indicated a discharge/charge capacity of 193/192 μAh at the 100th cycle, corresponding to a capacity retention of 99.5%. In particular, the HSi_HBP electrode can decrease the capacity by less than 1.5% compared with the value of the first cycle after 100 cycles, demonstrating excellent electrochemical stability.

## 1. Introduction

The powerful energy ability of LIBs enables their usage in numerous electric network applications, comprising improving the quality of energy harvested from wind, solar, geothermal, and other renewable sources, thereby expanding their usage and affording an energy-sustainable economy [1]. Therefore, LIBs have been widely used in many fields, such as portable electronics and electric vehicles, since their successful commercialization in the 1990s [2]. Furthermore, advanced LIBs have been used recently in the development of flexible LIBs [3]. The global market for flexible batteries was valued at USD 69.5 million in 2015 and is expected to reach USD 958.4 million by 2022 [4].

Developing components that satisfy performance conditions under external deformation stress is critical to the success of flexible energy sources. In addition to overcoming difficulties associated with flexibility, state-of-the-art flexible LIBs must address the challenging issues encountered by conventional LIBs, such as obtaining a high energy density, an improved rate capability, and new materials development [3]. When applying a new current collector, it should be in the form of a freestanding electrode, which can be obtained by either using electrically conducting materials that can form a film by itself or using a nonconducting flexible substrate on which an electrically conducting layer is coated. Carbon-based materials (carbon nanofibers, graphite sheets, carbon paper, carbon nanotubes (CNT), and graphene), conducting polymers, and flexible thin metals are potential candidates for flexible current collectors owing to their expected advantages [3]. Therefore, flexible LIBs with current collector requirements such as flexible, thin, lightweight, and sufficient electrical conductivity can be replaced by CNTs that satisfy these requirements simultaneously.

However, the use of theoretically high-capacity active materials to obtain high energy density batteries is inevitable, and the group IV elements (Si, Ge, and Sn) have been considered the most promising anode candidates for the next-generation LIBs [5,6]. Among these active materials, the theoretical capacity of the fully lithiated alloy Li_4.4_Si is 4212 mAh g^−1^ (10 times that of the commercial graphite anode), and Si primarily exhibits a long plateau in its discharge curve, providing a stable voltage during cycling. Furthermore, Si is not affected by solvent co-intercalation, affording an additional advantage over graphite [7]. However, the high storage capacity of Li atoms results in a severe volumetric change (>300%), resulting in the pulverization of electrodes and a subsequent loss of electrical contact between the Si-active material and the current collector [5,8]. These two problems result in a significant capacity decay in short cycles. In addition, the solid electrolyte interphase (SEI) formed on the Si anode surface due to the reduction of typically employed organic electrolytes is unstable. In this case, significant amounts of electrolytes and Li are consumed, and the distance of Li-ion diffusion is increased, resulting in a low Coulombic efficiency and material degradation [8]. Current methods of counteracting the aforementioned disadvantages associated with Si include the usage of Si nanowires, elaborate porous structures, and intricate C–Si composite structures [9]. CNTs are endowed with various useful properties, including a high aspect ratio, channels for lithium-ion intercalation, and excellent conductivity (electrical and thermal) [10]. The freestanding CNT electrodes can increase the usable anode specific capacity (Ah kg^−1^) in a battery by stored lithium as well as support ultrahigh capacity Si, which buffers the volume changes of active materials [5,10]. The integration of high electrical conductivity and flexible buckypaper (oxidized MWCNTs) and high-theoretical-capacity Si materials is effective for obtaining superior high-energy-density and flexible electrode materials [11]. Electrode preparation methods such as safety, environmental friendliness, low cost, and simplicity are important for the production of high capacity and lightweight flexible batteries. For example, Sarno et al. reported SC-CO_2_-assisted gel drying process was used to produce a porous solid electrolyte of PVDF-HFP able to uptake a high amount of ionic liquid and porous electrodes containing dispersed graphene oxide [12]. In order for the active material of the electrode to operate at a capacity close to that of sufficient theoretical value, it is necessary to find the interconnected network structure of the active materials during the preparation of the electrode [13].

Therefore, this study aimed to improve the electrochemical performance of randomly loaded integrated buckypaper (MWCN tubes) and a-Si (the thin-film Si buckypaper freestanding electrodes) using heat treatment as a simple and economical method for lightweight and flexible LIBs.

## 2. Materials and Methods

### 2.1. Preparation of Unheated Buckypaper

We prepared a freestanding flexible buckypaper for a Li-ion storage current collector using the filtration method. The experiments used MWCNTs produced in Carbon Nanomaterial Technology Co., Ltd., Pohang, Korea (~20 nm diameter, ~5 μm length, >95% purity, and <3% impurities).

**Figure 1:**
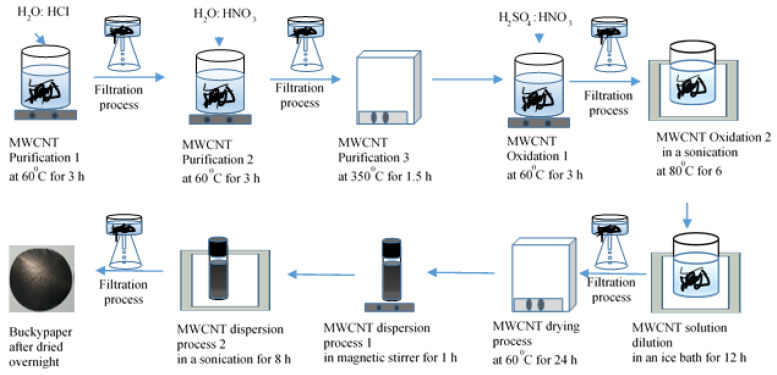
Schematic diagram of the unheated buckypaper preparation.

A schematic diagram of the unheated buckypaper preparation process via the wet method is shown in Figure 1. MWCNTs (0.5 g) were added to HCI (96%) with deionized (DI) water of 1:1 and then dispersed via mechanical stirring at 60 °C for 3 h to obtain a uniform solution. The purified MWNTs were filtered through a microporous membrane filter (pore size 0.45 μm) using filtration units with a vacuum pump and later washed until the pH of the solution was around 7. Subsequently, the second step of purification was performed, and the process was repeated for HNO_3_ (65%) with DI water (1:3 ratio) at 60 °C with magnetic stirring for 3 h instead of the previous process. The first two stages of purification were performed using HCI and HNO_3_ acids to remove metallic impurities that may form during MWCNTs production, followed by heat treatment to remove amorphous carbon after acid purification. The heat treatment process was continued at 350 °C for 2 h. Oxidation continued after the purification process. Concerning the use of CNTs as reinforcements in composite materials, the incorporation of oxygen-containing functionalities onto the graphitic surface is crucial for improving interfacial adhesion, i.e., the unique mechanical and electrical properties of CNTs can be transferred to the properties of CNT-based composites [14,15]. The oxidation process begins after the purification process. The oxidation process is a chemical reaction that combines oxygen-containing carboxyl groups in the MWCNT structure under the action of nitric and sulfuric acids, a mixture of strong acids, and combines the action of ultrasonic with heat to activate the reaction. The oxidation process took place in two stages, first, the purified MWCNTs were dispersed in 1:3 solutions of HNO_3_ (65%) and H_2_SO_4_ (98%) by using an ultrasonic bath with 60 °C for 6 h. After that, the resulting product was kept on the heater with a magnetic stirrer for 3 h. Then, the oxidized product was cooled thoroughly, and water was added until the volume increased 3 times, after which the soil was placed in an ice bucket and stored for 12 h. The oxidized MWNTs were filtered through a microporous membrane filter using filtration units and subsequently washed until the pH of the solution was around 7. The oxidized MWCNTs were dried in an oven at 60 °C for 24 h. The oxidized MWCNTs were subsequently dispersed in water, first by magnetic stirring for 1 h, followed by an ultrasonic bath for 3 h. To obtain a dense structure of MWCNTs, MWCNT dispersion was performed without heating, and the experimental temperature was con-trolled up to 25 °C. Subsequently, the resulting product was filtered and later dried for 24 h at room temperature. The next day, the resulting product was peeled off from the filtration membrane, and the resulting product is called buckypaper [14,15]. The thickness of the experimental buckypaper was adjusted to 17 μm, and the density was around 2.8 g cm^−1^.

### 2.2. Preparation of Heated Buckypaper and Silicon Buckypaper Electrodes via DC Magnetron Sputtering

Composite electrodes were created by depositing a thin film of Si on the resulting buckypaper via DC magnetron sputtering. A Si target was utilized as the active material in the current study. The Si target had a purity of 99.99% and a diameter of 50.8 mm. In our study, three types of electrodes were prepared in addition to the unheated buckypaper, depending on the type of active materials, such as buckypaper and Si heat treatment. The electrode was fabricated in three primary steps. To illustrate the effect of heat treatment, the electrodes were labeled as UBP (Unheated_buckypaper), HBP (Heated_buckypaper), USi_HBP (heated buckypaper and Si thin film without heat treatment), and HSi_HBP (both buckypaper and Si thin film with heat treatment).

**Figure 2:**
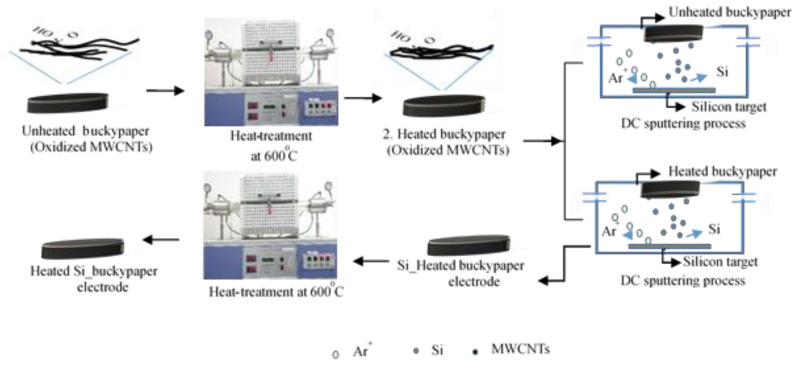
Schematic diagram of the electrode preparation (HBP, USi_HBP, and HSi_HBP)**.**

A schematic diagram of the preparation process is shown in Figure 2 For the heated buckypaper electrode; the unheated buckypaper was heat-treated at 600 °C for 1.5 h in a vacuum-sealed quartz tube. For the HSi_HBP electrode, a-Si thin film was deposited on heated buckypaper substrates via DC magnetron sputtering. The Si thin film was deposited at room temperature, the base pressure in a vacuum was 5 × 10^−6^ Torr, and the deposition pressure was maintained at 2 × 10^−3^ Torr. The Ar gas flow rate was 20 sccm, and the deposition rate of the film was 20 nm/min. The Si target was pre-sputtered for 15 min, and the deposition time was 15 min at 200 W DC power. Regarding the HSi_HBP electrode, before commencing the coating process, the unheated buckypaper was heat-treated at 600 °C for 0.5 h in a vacuum-sealed quartz tube. Subsequently, the Si thin film was deposited with the heated buckypaper under the same sputtering conditions. Next, the Si thin film on the HBP electrode was reheated at 600 °C for 1 h in a vacuum-sealed quartz tube after the Si sputtering process.

### 2.3. Characterization of Electrode

The structures of silicon thin films and buckypapers before and after heat treatment were investigated via X-ray diffractometry (XRD, Rigaku, Miniflex, Tokyo, Japan, Cu Ka) and Raman scattering spectroscopy (RAMAN, HORIBA, LabRAM HR800, Tokyo, Japan, laser excitation at 532 nm) in the frequency range of 50–3000 cm^−1^. The morphologies of the deposited films and buckypapers before and after heat treatment were determined via field emission scanning electron microscopy (FE-SEM, JEOL, JSM-6701F, Tokyo, Japan). The pour-probe technique was used to investigate the electrical conductivity of the buckypapers before and after heat treatment. Flat and bent coin cell types CR 2032 were assembled in an Ar-filled glove box. Li metal foil was used as the counter and reference electrodes, a microporous polypropylene membrane as the separator, and a 1 M solution of LiPF_6_ in ethylene carbonate: diethyl carbonate (1:1, vol.%) as the electrolytic solution. Galvanostatic charge-discharge tests were performed on the flat and bending coin cells using a battery cycler (WonATech, WDCS3000s, Seoul, Korea) at various current densities at a voltage ranging between 0.01 and 2 V. Cyclic voltammetry (CV) analyses were performed using a Gamry Instrument(ZIVELAB, ZIVEsp2, Seoul, Korea) over the potential range of 0.01–2.0 V. The dynamic process of discharge/charge transformation in lithiation/delithiation was observed via electrochemical impedance spectroscopy in the frequency range of 1 to 10 MHz with a cutoff of 0.15 V. The electrode bending test was performed on a bending stage machine for different durations at a constant radius of 8.3 mm.

## 3. Results

### 3.1. Characterization of Electrodes

**Figure 3:**
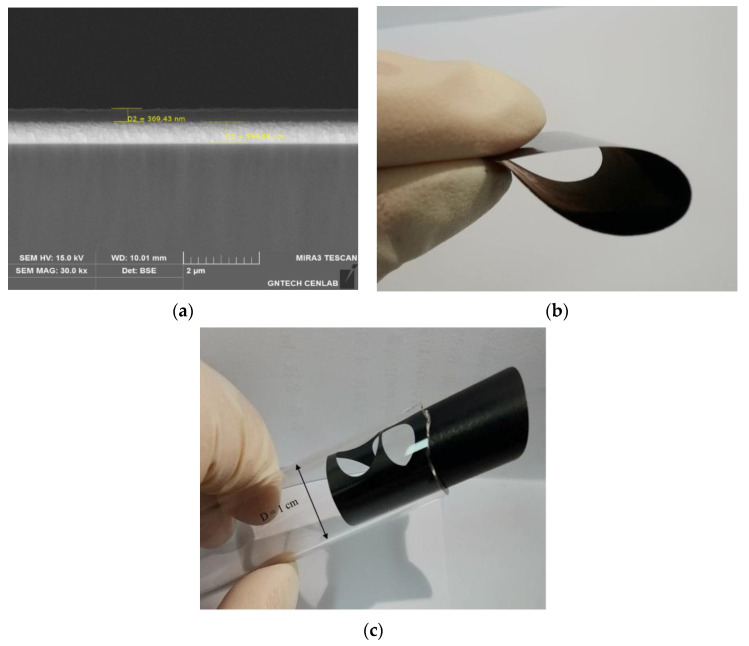
Cross-sectional view of (**a**) Si deposited on the titanium and photograph of free-standing flexible (**b**) UBP and (**c**) HBP being rolled (3 × 3 mm^2^).

Figure 3a shows a cross-sectional view of the deposited Si. To accurately determine the thickness of the stored silicon, the silicon had to be stored on a flat surface, and we stored the titanium on the slide glass and then stored the silicon on the titanium to create suitable measurement conditions. The thickness of the deposited Si was 0.369 μm, while the deposition time of the sputtering process was 15 min.

The amount of the deposited Si was calculated using the formula m = ρsd, where s and d represent the thickness and area of the electrode, respectively, and ρ is the density of the active material, which was assumed to be the theoretical density (of 2.26 ± 0.1 g cm^−3^) for amorphous silicon [16,17]. The weights of the buckypaper before and after heat treatment were measured using an analytical balance. The buckypaper lost approximately 13% of its total weight after heat treatment because of a 5% loss associated with the evaporation of water absorbed from the walls of the CNT at temperatures of 180 °C, whereas the remaining weight loss corresponded to the elimination of hydroxyl groups and the oxidation of amorphous carbon and impurities at 400–500 °C [18,19]. The electrical conductivities of UBP and HBP were measured using the four-probe technique. The electrical conductivity of the UBP was 0.1 × 10^5^ S/m at room temperature, whereas that of the HBP was 0.16 × 10^5^ S/m at 600 °C. The buckypapers indicated similar electrical conductivity values, although that of the HBP increased slightly, indicating that the electrical conductivity was dependent on temperature because electrons gain sufficient energy to move freely from the valence band to the conduction band, thereby reducing the internal resistance [20].

The electrical conductivities of the electrodes are shown in Table 1, along with the average thicknesses and weights of the electrodes. Figure 3 shows a photograph of (b) a freestanding flexible UBP and that of (c) a rolled (3 × 3 mm^2^) HBP. The buckypaper is extremely flexible, retains its original shape without any damage when folded or rolled, and can be rolled up to 10 mm (when determining the diameter of the buckypaper wrapped in a circle, the diameter is rolled up to 10 mm). The flexibility of buckypaper is maintained after heat treatment and it can bend down in a 10 mm tube after heat treatment, as shown in Figure 3c. The flexible properties of buckypapers before and after heat treatment for bending and roll-up did not indicate any significant difference.

**Figure 4:**
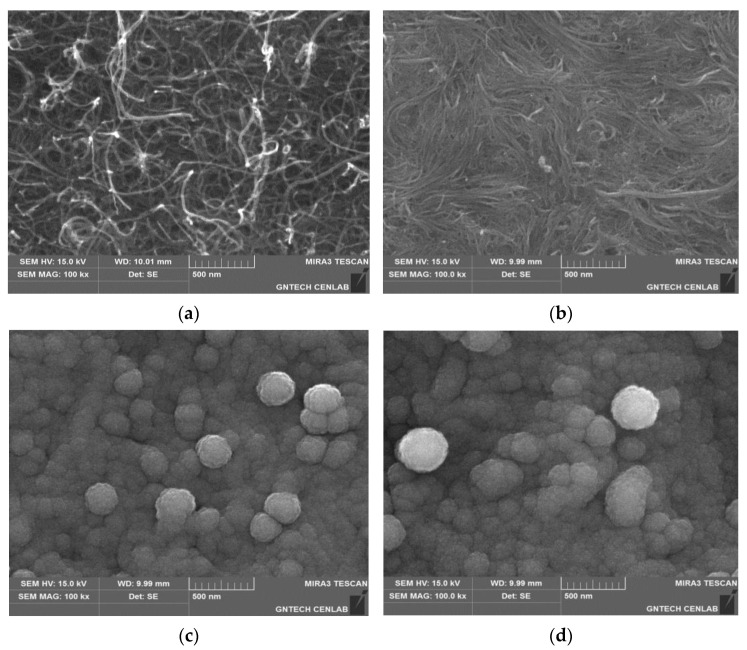
SEM images of the buckypapers (**a**) before and (**b**) after heat-treatment; and those of (**c**) USi_HBP and (**d**) HSi_HBP.

Figure 4 shows the SEM images of the buckypaper (a) before and (b) after heat-treatment, as well as those of the (c) USi_HBP, and (d) HSi_HBP. As shown in Figure 4a,b, after heat treatment, the MWCNTs in the buckypaper structure was compressed and the free space was reduced to a denser structure [21]. It has been reported that owing to heat treatment, the specific surface area of buckypaper decreased from 286.5 to 252.1 m^2^/g, whereas the amorphous region and the nonreacted catalysts or impurities were removed from the unheated CNTs [21]. As shown by the SEM images, the same trend was observed for the USi_HBPand HSi_HBP electrodes in terms of grain size. The grain size of the USi_HBP electrode was 149 nm and 141 nm for HSi_HBP Figure 4c,d.

**Figure 5:**
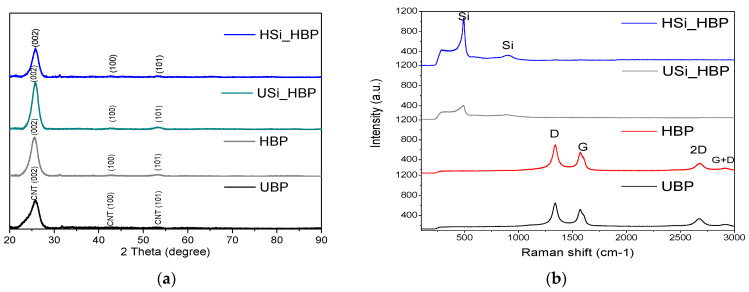
(**a**) XRD patterns and (**b**) Raman spectra of UBP, HBP, Si_HBP, and HSi_HBP electrodes.

As shown in Figure 5, the electrodes were characterized through XRD and Raman spectroscopy to analyze the changes in their crystalline qualities (perfection) as a result of the heat treatment. In the XRD results (Figure 5a), a broad substrate of ((002), (100), (101)) was observed in all electrodes [20,21,22,23,24,25,26,27]. All diffraction peaks were observed only of the CNTs owing to the heating time of 1 h and did not disrupt the structural properties of the buckypaper and amorphous Si, consistent with the reported data. No characteristic peaks were observed for the other impurities. In the buckypaper before and after heat treatment, the peaks for the D, G, 2D, and D + G-bands of the Raman spectrum of CNT appeared at wavelengths near 1341 cm^−1^, 1571 cm^−1^, 2670 cm^−1^, and 2704 cm^−1^, respectively [21,28,29,30]. The 2D and G bands were present in all carbon samples, and the D band appeared because of the disorder-induced in sp^2^-bond carbon, whereas the G band was due to the in-plane vibration of the sp^2^ carbon atoms [30]. The intensity ratio of the G to D bands (IG/ID) can be used to evaluate the degree of crystalline perfection [21]. The IG/ID ratio of the Heated buckypaper was 0.79, which is similar to that of the UBP of 0.81. Therefore, the Raman result confirms that no crystallization was observed on the HBP after heat treatment. For the USi_HBP and HSi_HBP electrodes, three characteristic broad peaks centered at 481 and 795 cm^−1^ were assigned to amorphous Si [31,32,33,34], whereas the D and G-bands of the CNT disappeared, indicating that the surface of the carbon electrode was covered by amorphous Si [34]. The Raman spectrum showed that the Si was still in the amorphous state, and a crystalline Si peak did not appear. These results agreed well with those from a previous study, in which both the process temperature and time were important parameters for Si crystallization (sharp crystalline Si peak at 524 [33]) [35]. Another study reported that the nucleation of crystalline silicon occurs at a temperature of 650 °C [36].

### 3.2. Electrochemical Characterization of Electrodes

**Figure 6:**
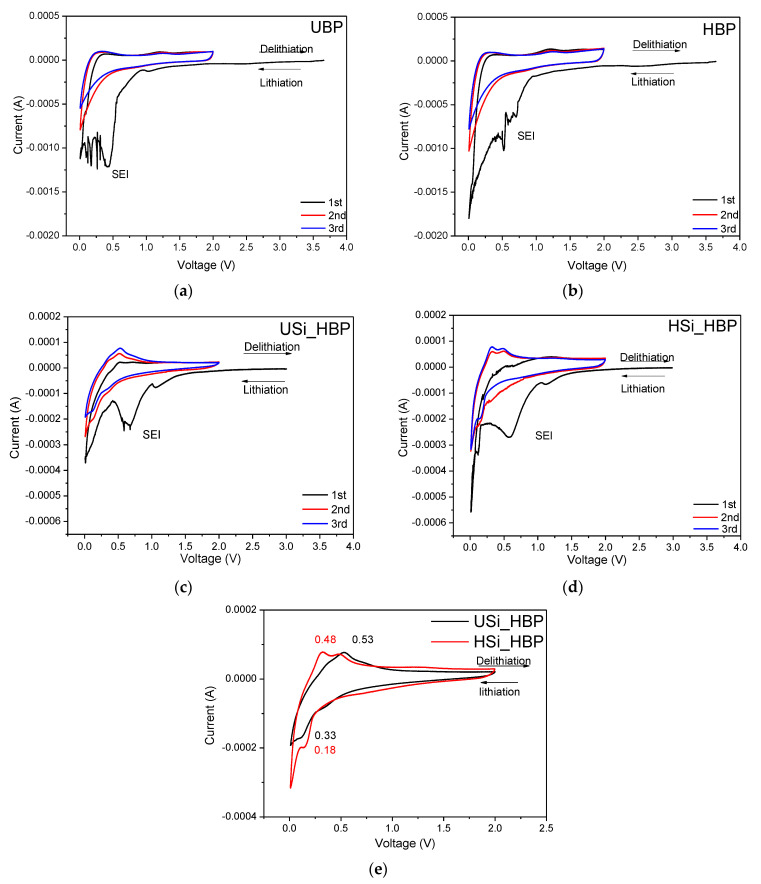
The cyclic voltammograms of (**a**) the UBP, (**b**) HBP, (**c**) USi_HBP, and (**d**) HSi_HBP electrodes; (**e**) third CV curves of both Si electrodes at a 0.1 mV s^−1^ scanning rate between 0.01 and 2.0 V.

Figure 6 shows the CV of the (a) UBP, (b) HBP, (c) USi_HBP, and (d) HSi_HBP electrodes. CV measurements were performed for the first three cycles within a voltage window of 0.01–2.0 V at a scanning rate of 0.1 mV s^−1^.

During the first cycle, both buckypapers exhibited cathodic peaks at 0.4–0.75 V, which can be attributed to the SEI formation on the surface of the CNT film electrode (Figure 6a,b) [20,21,37,38]. These peaks in the first cycle during the reduction process did not form in the subsequent cycle owing to the stability of the SEI formation during the first cycle [21,37,38]. The anodic peak at approximately 0.4 V was introduced by the delithiation of the CNT film electrodes [21] corresponding to the reaction of LiC_6_ (LiC_6_ → C_6_ + Li^+^ + e^−^) [34,35]. The intensity of the peaks in the HBP was lower than that of the UBP, which may reduce the surface area after heat treatment and reduce the SEI formation, resulting in limited lithium inclusion [21,39]. The anodic and the cathodic voltage curves of the HBP shifted slightly toward the negative and positive directions, implying changes in the electronic resistance [20,21]. The CV curves of the USi_HBP and HSi_HBP show that both the heat-treated electrodes exhibited typical redox features of Si with lithium insertion occurring at potentials below 0.3 V and extraction of lithium ions from Li-Si alloys at 0.36 and 0.53 V (Figure 6c,d, respectively) [40,41,42,43,44,45,46]. For both electrodes, broad cathodic peaks appeared at 0.61 and 1.2 V in the first cycle but disappeared in the following cycles, indicating an irreversible reaction due to the formation of an SEI layer [5,41]. For both electrodes, from the second to third cycles, two cathodic peaks around 0.21 V and 0.01 V appeared, corresponding to the formation of Li_x_Si alloy phases, accompanied by an increased peak intensity, which indicated a further degree of alloying with the increasing number of cycles [41]. Figure 6e shows a comparison of the third CV curves for the electrodes of USi_HBP and HSi_HBP to further illustrate the change in the curves. The oxidation and reduction peaks of the USi_HBP electrode were observed at 0.53 (0.38) and 0.15 V, respectively, whereas they were observed at 0.48 (0.33) and 0.18 V for the heated Si_buckypaper electrode, respectively. The peak potential difference between the oxidation and reduction peaks was 0.3 V for the HSi_HBP and 0.38 V for the USi_HBP, indicating less polarization in the HSi_HBP electrode. In addition, a higher peak indicates higher Li-ion diffusion and lower internal resistance [20,21]. Based on these results, the resistance of the HSi_HBP electrode decreased because of an increase in hydrogen concentration and fewer dangling bonds after the treatment of Si (from 200 °C) [47,48].

**Figure 7:**
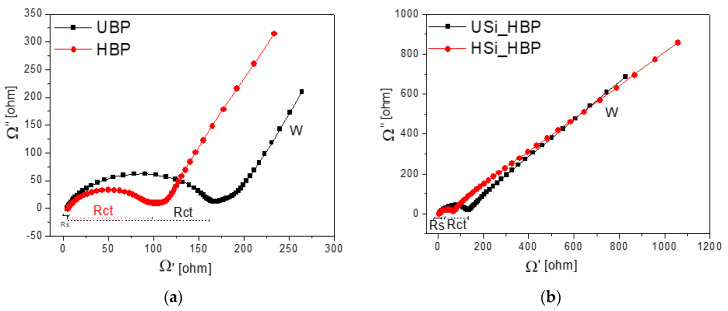
Electrochemical impedance spectra of the (**a**) buckypapers and the (**b**) heat-treated Si_buckyaper electrodes at fifth discharge cycles at a frequency range of 1 to 10 MHz with cutoff of 0.15 V.

Figure 7 shows a comparison of the Nyquist plots of the electrodes to provide a better understanding of the charge transfer and ion transfer mechanism of the electrodes. The Nyquist plots were obtained at a frequency range of 1 MHz to 10 mHz with 0.15 V in the discharged state at the fifth cycle. The Nyquist plot exhibited a suppressed semicircle in the high-to-medium frequency region and a diagonal straight line at low frequencies; the former is ascribed to charge transfer, whereas the latter is attributed to the lithium-ion diffusion of the electrode material [41,49,50]. The Nyquist plots show solution resistance (R_s_) from zero to the beginning of the semicircle and charge transfer resistance (R_ct_) up to the end of the semicircle [51]. As shown in Figure 7a, the solution resistances were similar, i.e., 6.3 Ω for the UBP and 4.3 Ω for the HBP electrode, whereas the semicircle radius of the HBP electrode (R_ct_ = 101 Ω) was much smaller than that of the UBP electrode (R_ct_ = 169 Ω). These curves demonstrate that the impedance in the electrode decreased, the insertion and delithiation of Li ions became easier, and the electrochemical performance improved [21]. Based on the Si electrodes shown in Figure 7b, the diameter of the semicircle of the HSi_HBP (63 Ω) was smaller than that of the USi_HBP electrode (125 Ω), indicating a much lower cell impedance. This lower impedance enabled the cells to decrease their polarization and maintain stable electrochemical states in terms of cycle stability and rate capability [45,49,51]. The reaction mechanism of lithiation and delithiation of a single buckypaper electrode differs from that of a silicon-coated buckypaper, and in this study, the capacity of the electrodes is expressed in milliampere-hours.

**Figure 8:**
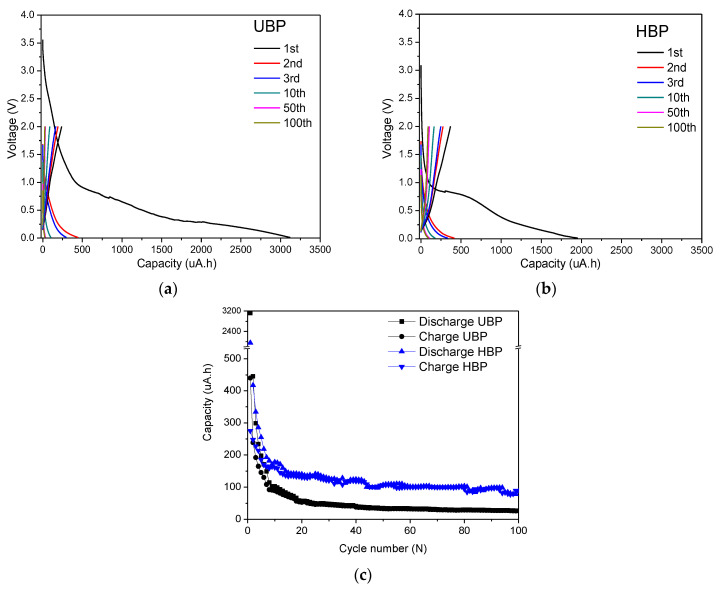
Charge/discharge capacity profile of (**a**) UBP and (**b**) HBP; discharge and charge cycling performance of (**c**) buckypaper electrodes with a current density of 125 μA and cutoff voltage of 0.01–2.0 V.

The charge/discharge capacity profiles of the (a) UBP and (b) HBP as well as the discharge and charge cycling performances of (c) the buckypaper electrodes with a current density of 125 μA and cutoff voltages of 0.01–2.0 V are shown in Figure 8. The UBP and HBP electrodes showed superior initial discharge capacities (3121 and 1955 μAh, respectively) that were higher than the theoretical capacity of MWCNTs, indicating the existence of additional lithium storage sites in the first cycle [20]. The initial discharge/charge capacities of the UBP were 3121/238 μAh, respectively, corresponding to a Coulombic efficiency of 8%, whereas those of the HBP were 1955/368 μAh, respectively, corresponding to a Coulombic efficiency of 19%. In addition, based on the CV test profile, the same plateau range of both electrodes from 0.8 to 0.01 V in the first cycle indicated SEI layer formation [52]. This large capacity loss was attributed to the large active surface area of the CNT, which increased the amount of Li involved in the formation of the SEI [21]. Additionally, the CNTs demonstrated a greater loss of Li ions because Li+ was irreversibly intercalated within the inner core of the nanotubes, further worsening the irreversible capacity loss [21,26,53]. The HBP had a relatively high Coulombic efficiency in the first cycle compared with the UBP owing to the reduced amount of lithium-ion residue due to the formation of a denser structure and improved bonding between the MWCNTs, as shown in the SEM images. During the first discharge process, the HBP showed a much smaller discharge capacity than the UBP, which can be explained by the reduction of the lithium-ion delivery area due to the reduction of the free space of the UBP due to heat treatment. Beginning from the second cycle, the capacitance of the two electrodes began to stabilize, and at the second and fifth cycles, the discharge/charge of the UBP were 445/192 μAh and 27/26 μAh, respectively, corresponding to Coulombic efficiencies of 43% and 96%, respectively. For the HBP, the discharge/charge was 417/275 μAh and 95/93 μAh at the second and fifth cycles, respectively, corresponding to Coulombic efficiencies of 66% and 98%, respectively. In terms of the cycle performance of the buckypapers (Figure 8c), the HBP maintained a higher capacity than the UBP electrode for 100 cycles, and the capacity retention after the 100th cycle was 11% for the UBP and 25% for the HBP. The improvement in the HBP at high capacities and the cycling retention contributed to nonreacted catalysts or impurities; furthermore, larger pore sizes of UBP were removed, and the MWCNT tubes formed denser structures [21].

**Figure 9:**
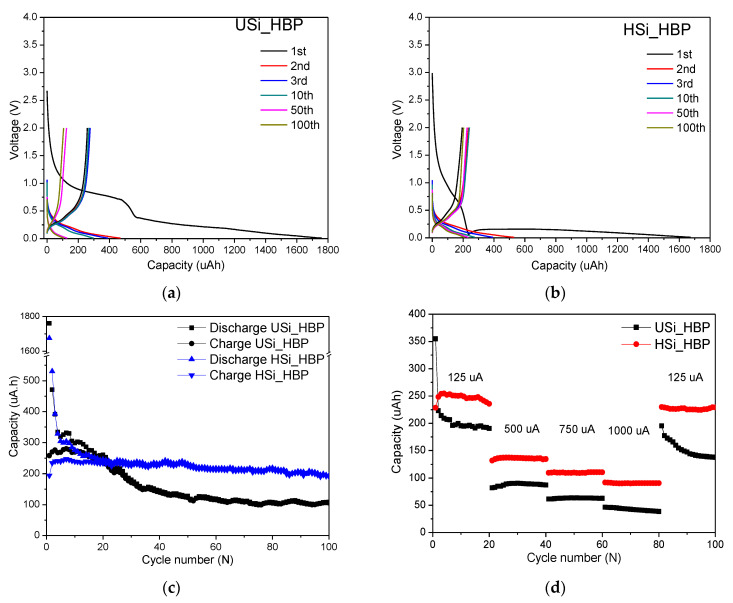
Charge/discharge capacity profiles of (**a**) USi_HBP and (**b**) HSi_HBP, (**c**) discharge/charge cycling performance of electrodes at a current density of 125 μA with the voltage of 0.01–2.0 V, and (**d**) charge cycling performance of heat-treated Si electrodes at progressively increasing current densities from 125 to 1000 μA.

The charge/discharge profiles of the USi_HBP and HSi_HBP electrodes, presented in Figure 9a,b, respectively, were between 0.01 and 2.0 V at 125 μA. The measured initial discharge/charge capacities of the USi_HBP electrode were 1761/258 μAh, corresponding to a Coulombic efficiency of 15%, whereas the HSi_HBP electrode indicated an initial discharge/charge capacity of 1675/194 μAh, corresponding to a Coulombic efficiency of 12%. Both electrodes demonstrated extremely low Coulombic efficiencies in the first cycle. The low Coulombic efficiency of the first cycle can be explained by the high concentration of MWCNTs (97.9 wt.%), which was the main component of the electrode [46,54]. This initial irreversible capacity loss is attributed to Li loss due to the formation of an SEI layer [5,54]. Furthermore, the USi_HBP electrode delivered a high discharge/charge capacity of 471/269 μAh in the second cycle, and the Coulombic efficiency recovered and increased to 90% by the 10th cycle. For the HSi_HBP electrode, the discharge/charge capacities were 530/234 μAh in the second cycle and 275/245 μAh on the 10th cycle, corresponding to a Coulombic efficiency of 89%. Regarding the comparison of cycling performance shown in Figure 9c, the USi_HBP indicated a higher capacity until the 20th cycle; subsequently, the capacity slowly decreased in the following cycles, and the capacity became 108/106 μAh, corresponding to a capacity retention of 41% at the 100th cycle. For the HSi_HBP electrodes, the capacity was maintained for 100 cycles, which delivered high capacities of 193/192 μAh at the 100th cycle, and superior cycling stability, with 99.5% capacity retention after 100 cycles. The wide electrode expansion of Si was relieved, and the two electrodes showed excellent electrochemical performance, indicating that the unique surface properties of the MWCNTs can easily buffer the stress arising from volume increment [19]. The two electrodes demonstrated extremely high capacity, indicating that the heat treatment of buckypaper can triple the capacity (Figure 7). The improved performance of the HSi_HBP electrode (an extremely stable cycling performance) can be attributed to the reduced hydrogen content in the amorphous silicon structure and the hanging bond, as well as factors such as internal stress after the heating process (Figure 7) [36,47,48]. Furthermore, the rate capability of the electrodes was probed by applying different current densities ranging from 125 μA to 1000 μA (as shown in Figure 9d). The charge capacities after 20 cycles were 191, 88, 63, 39, and 138 μAh at the current densities of 125, 500, 750, 1000, and 125 μA for the USi_HBP, respectively, whereas those of the HSi_HBP were 235, 139, 111, 91, and 229 μAh, respectively. Hence, the two electrodes were able to maintain their capacity at each step, and the capacity decreased as the charging rate increased. It has been reported that at high current densities, the proportion of side reaction increases as a direct consequence of the Bulter-Volmer equation, resulting in a nonreversible reaction and momentarily decrease in capacity [20]. In addition, the motion of Li ions was rapid, preventing the entire active material to participate in initial cycles [20]. Consequently, HSi_HBP indicated a higher capacity than the USi_HBP at each current density. However, the two electrodes indicated good recovery performance when the current rate returned to 125 μA, in which the electrodes exhibited an excellent stable structure with a stable electrical contact [46]. Electrochemical results show that a simple method of heat treatment allows a properly regulated structure of the electrode to be obtained and can replace chemicals such as additional binder, which is responsible for tightly bonding the active materials.

**Figure 10:**
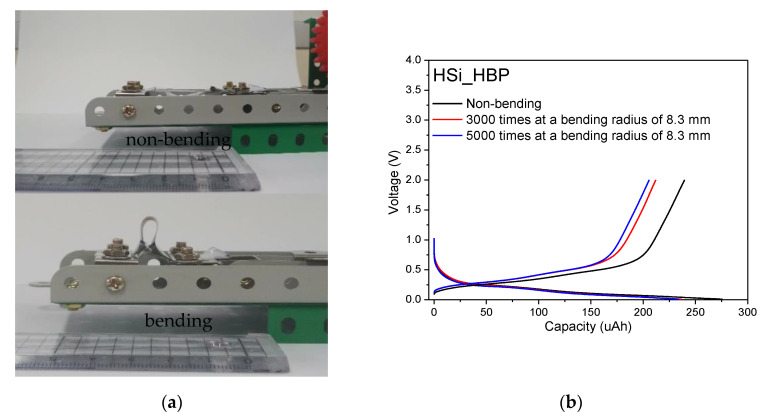
Bending-state electrochemical testing of HSi_HBP electrodes. (**a**) Photograph of bending test for 8.3 mm radius at two different durations; (**b**) Charge/discharge capacity profiles of electrodes at a current density of 125 μA with the voltage ranges of 0.01–2.0 V.

Buckypaper fabricated using MWCNT with Si has been reported to exhibit flexible properties and hence can be used in flexible batteries [5]. Our buckypaper in this study comprised oxidized MWCNT and exhibited flexible properties (Figure 3). Therefore, we performed a bending test for a flexible device; the bending test was performed using a bending stage machine at two different repetitions of 3000 and 5000 at a constant 8.3 mm radius and a speed of 35 times min^−1^. After performing the bending test as shown in Figure 10a, the half-cell was assembled at a current density of 125 μA in the voltage range of 0.01–2.0 V. The charge/discharge capacity profiles of the bent HSi_HBP electrodes at the third cycle and the platform of the HSi_HBP electrode are shown in Figure 10b.

The non-bent electrode and electrodes bent for 3000 and 5000 times showed third discharge/charge capacities of 275/240 μAh with a Coulombic efficiency of 87%, 236/212 μAh with a Coulombic efficiency of 89%, and 234/206 μAh, respectively, with a Coulombic efficiency of 88%. Compared with the non-bent electrode, the discharge capacity losses of the electrode bent 3000 and 5000 times were 14.2% and 14.9%, respectively. Although the capacities of the bent batteries were not exactly the same as those of the non-bent battery, they exhibited excellent energy density compared with the batteries that did not use buckypaper (MWCNTs); furthermore, their energy density can be increased. Our findings showed that the heat-treated Si_buckypaper electrodes exhibited high capacity, high Coulombic efficiency, and good cycling stability, offering the promising potential for applications in flexible batteries.

## 4. Conclusions

In this study, an independent flexible electrode was successfully assembled by oxidizing MWCTs, and depositing a-Si thin film on the buckypaper was produced via magnetron sputtering. The structural properties and quality of the active materials (CNTs and thin-film silicon) were confirmed via SEM, XRD, and Raman studies, and the results confirmed that the electrode materials exhibited the same properties before and after heat treatment. The results of electrochemical show that buckypaper contributes to the storage of lithium ions, and after heat treatment, the storage capacity can be tripled. The electrochemical results show that in the case of a-Si, the ability to store a-Si without heat is insufficient, while heat treatment can dramatically improve the ability to store Li^+^. The ability of this HSi_HBP electrode in retaining its capacity can be attributed to the reduction in hydrogen concentrations and dangling bonds in the a-Si structure due to heat treatment, resulting in a more stable structure, improved bonding of active materials, reduced internal stress, and better charge transfer. The results of the bending test prove that electrodes can be used in flexible batteries, and the effect of heat treatment is a positive approach to any of the properties of the electrode without compromising the flexibility of the electrode. Here, we highlight a simple heat treatment method that can dramatically improve the electrochemical properties of high-capacity flexible buckypaper silicon electrodes and show that heat-treated electrodes can store energy in full compliance with modern technological developments at low cost and with flexibility and excellent performance.

## Figures and Tables

**Table 1:** Characteristics of UBP and HBP electrodes.

Sample	Average Thickness (μm)	Weight (mg)	Electrical Conductivity (S m^−1^)
UBP (at room temperature)	17	2.8	0.10 × 10^5^ ± 100
HBP (at 600 °C)	17	2.8	0.16 × 10^5^ ± 100

## Data Availability

Data Sharing is not applicable.
